# Supplementary material for: A Highly Sensitive and Flexible Capacitive Pressure Sensor Based on Alignment Airgap Dielectric
Source: Sensors (Basel). 2022 Sep 28;22(19):7390. doi: 10.3390/s22197390 (PMC9571520; doi:10.3390/s22197390)
Supplement: Supplementary file 1 [file sensors-22-07390-s001.zip › sensors-1884235-supplementary.pdf]

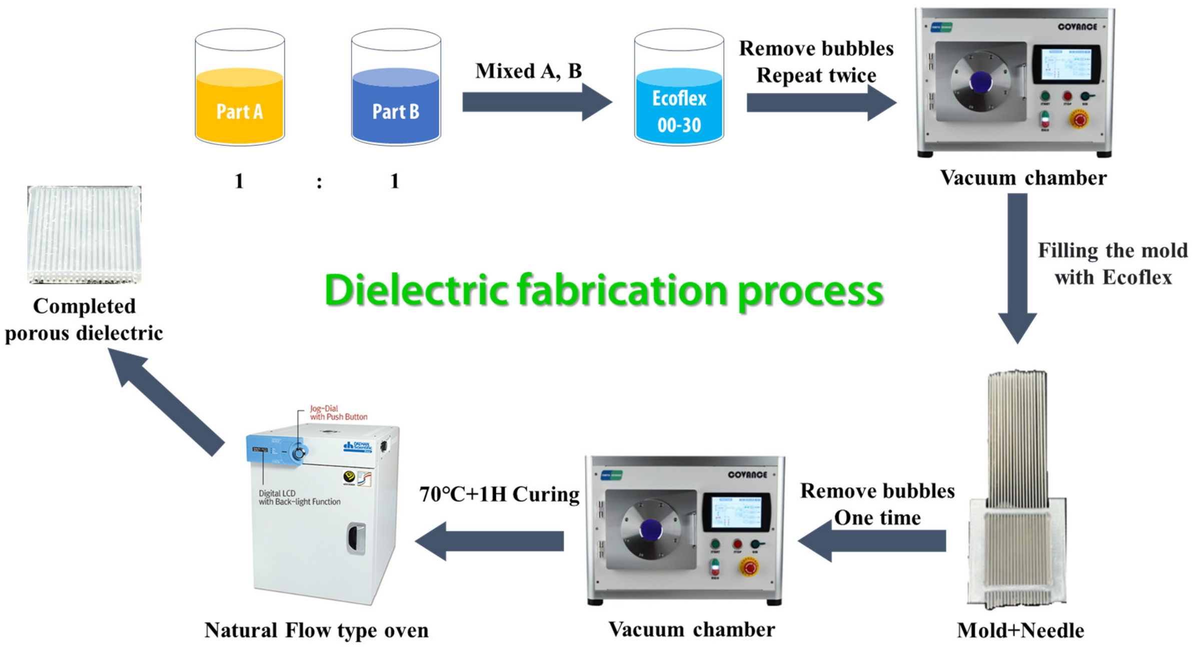

Figure S1. Fabrication process of Alignment airgap-based capacitive pressure sensor.

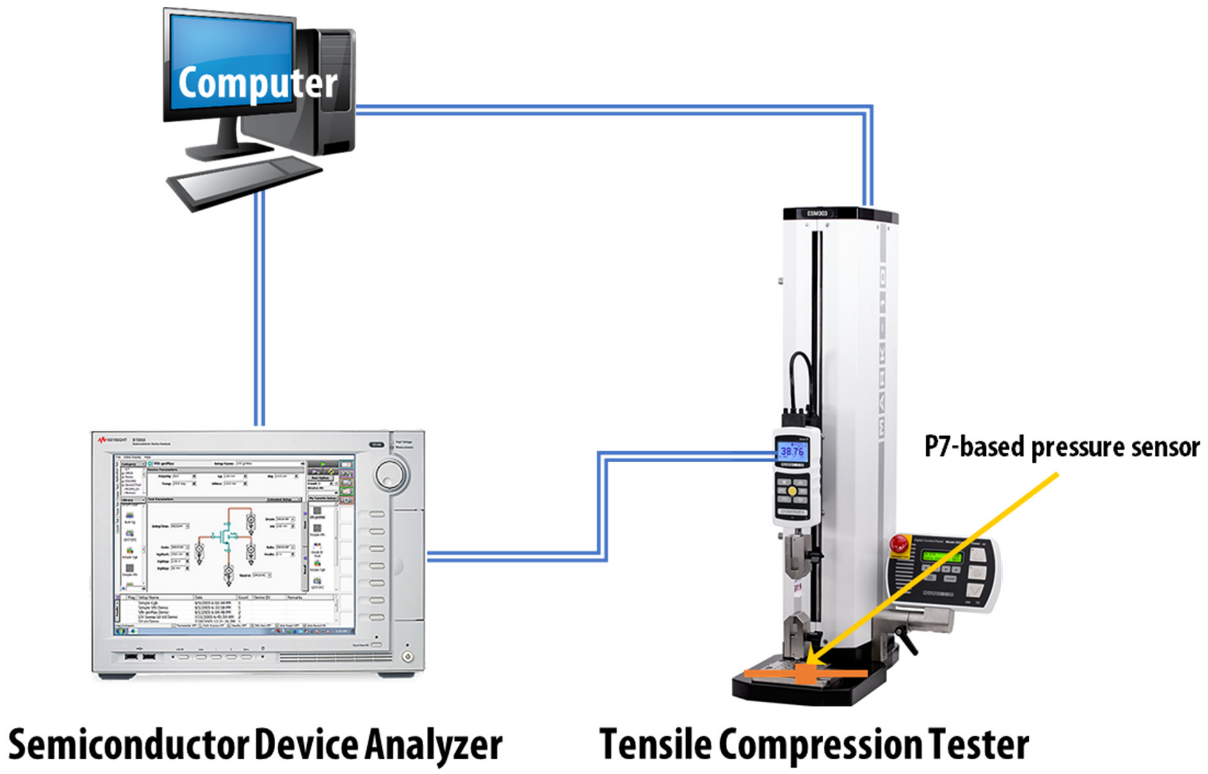

Figure S2. Schematic of the alignment airgap-based capacitive sensor testing set-up.

Table S1. Performance comparison of the alignment airgap-based sensor fabricated in this research and precedent research.

Material/Structure	Sensitivity (Pressure range)	Response/ Recover time	Limit of detection	Reference
Porous PDMS	0.18 kPa^{-1}(0-400kPa)	100 ms	10 Pa	[34]
PDMS/DIW	0.068 kPa^{-1}(0.01-0.05 kPa)<br>0.095 kPa^{-1}(0.1-0.5 kPa)	110 ms	1 Pa	[38]
Porous PDMS	0.813 kPa^{-1}(< 0.2 kPa)	70 ms	0.118 g	[39]
Micro-arrayed PDMS	2.04 kPa^{-1}(0-2 kPa)	100 ms	7 Pa	[40]
Porous PDMS	0.63 kPa^{-1}(< 1 kPa)	40 ms	2.42 Pa	[41]
Alignment Airgap Ecoflex	1.277 kPa^{-1}(≤ 0.5 kPa)	100 ms	20 Pa	This work
